# Kinetic Energy Dose as a Unified Metric for Comparing Ball Mills in the Mechanocatalytic Depolymerization of Lignocellulose

**DOI:** 10.3389/fchem.2021.816553

**Published:** 2022-01-03

**Authors:** Martin Kessler, Roberto Rinaldi

**Affiliations:** Department of Chemical Engineering, Imperial College London, London, United Kingdom

**Keywords:** mechanocatalysis, mechanochemistry, biorefinery, lignocellulose acid-hydrolysis, depolymerization, kinematic model, ball mill, energy dose

## Abstract

Mechanochemistry utilizes mechanical forces to activate chemical bonds. It offers environmentally benign routes for both (bio) organic and inorganic syntheses. However, direct comparison of mechanochemistry results is often very challenging. In mechanochemical synthetic protocols, ball mill setup (mechanical design and grinding vessel geometry) in addition to experimental parameters (milling frequency, duration, ball count and size) vary broadly. This fact poses a severe issue to further progress in this exciting research area because ball mill setup and experimental parameters govern how much kinetic energy is transferred to a chemical reaction. In this work, we address the challenge of comparing mechanochemical reaction results by taking the energy dose provided by ball mills as a unified metric into account. In this quest, we applied kinematic modeling to two ball mills functioning under distinct working principles to express the energy dose as a mathematical function of the experimental parameters. By examining the effect of energy dose on the extent of the mechanocatalytic depolymerization (MCD) of lignocellulosic biomass (beechwood), we found linear correlations between yield of water-soluble products (WSP) and energy dose for both ball mills. Interestingly, when a substrate layer is formed on the grinding jar wall and/or grinding medium, a weak non-linear correlation between water-soluble products yield and energy dose is identified. We demonstrate that the chemical reaction’s best utilization of kinetic energy is achieved in the linear regime, which presents improved WSP yields for given energy doses. In the broader context, the current analysis outlines the usefulness of the energy dose as a unified metric in mechanochemistry to further the understanding of reaction results obtained from different ball mills operating under varied experimental conditions.

## Introduction

Mechanochemistry employs mechanical forces to drive chemical reactions, which can be sustained by milling, grinding, or extrusion with no need for bulk dissolution of reactants, thus opening up new avenues for the design of organic reactions (Stolle et al., 2011; Do and Friščić, 2016; Hernández and Bolm, 2017). Importantly, mechanochemistry enables chemical reactivity inaccessible in solution. In this instance, solvent-free catalytic routes to depolymerize cellulose (Hick et al., 2010; Meine et al., 2012; Hilgert et al., 2013; Shrotri et al., 2013; Zhang and Jerome, 2013; Käldström et al., 2014a; Liao et al., 2014; Schüth et al., 2014; Yabushita et al., 2014; Dornath et al., 2015; Shrotri et al., 2015; Dong et al., 2016; Schneider et al., 2016; Yu et al., 2016; Schneider et al., 2017b; Furusato et al., 2018; Karam et al., 2018; Kobayashi and Fukuoka, 2018), lignocellulosic biomass (Meine et al., 2012; Carrasquillo-Flores et al., 2013; Käldström et al., 2014a; Käldström et al., 2014b; Schüth et al., 2014; Loustau-Cazalet et al., 2016; Schneider et al., 2017a), and more recently, technical lignins (Kleine et al., 2013; Dabral et al., 2015; Brittain et al., 2018; Dabral et al., 2018) constitute outstanding examples demonstrating the potential of mechanochemistry to enable chemical reactivity inaccessible in solution under ambient conditions. These routes are known as mechanocatalytic depolymerization (MCD). Recently, atomistic modeling provided in-depth insight into the impact of mechanical forces on the MCD of cellulose and lignin (Amirjalayer et al., 2019). On the one hand, mechanical forces are proposed to produce conformational changes in the pyranic ring, activating latent states in the MCD of cellulose, facilitating the glycosidic bond cleavage (Schüth et al., 2014). In this manner, compared to a thermally activated process, the mechanochemical conversion was predicted to decrease the activation barrier by a factor of five (Amirjalayer et al., 2019). On the other hand, the cleavage of the most common lignin linkages (*β*-O-4 aryl alkyl ether) seems to be favored by stretching forces (Patel et al., 2020). Stretching of polymeric lignin chains may also be achieved by friction in a ball mill.

Experimentally, MCD of lignocellulose is performed by subjecting dry (ligno)cellulosic biomass, impregnated with strong acids (*e.g.*, H_2_SO_4_, HCl, *p*-toluenosulfonic acid) or mixed with solid acids (*e.g.* kaolinites), to ball milling for a duration of 1–3 h. Under optimized conditions, this procedure fully converts lignocellulosic materials into water-soluble products (WSP) *via* acid-catalyzed depolymerizing reactions on cellulose, hemicellulose, and lignin (Hick et al., 2010; Meine et al., 2012). Other chemical processes also happen together with MCD, contributing to the water solubility of the reaction products. Notably, the formation of α(1→6)-oligoglucosaccharides from cellulose and hemicellulose enhances the water solubility of the products (Shrotri et al., 2013; Dornath et al., 2015). In addition, native lignin also undergoes depolymerization (Calvaruso et al., 2017b). Therefore, WSP obtained from lignocellulose is a complex mixture of oligosaccharides and depolymerized lignin oligomers. Under low severity conditions (pH 1, 140°C for 1 h), the WSP can easily undergo further saccharification in aqueous medium, leading to lignin precipitation. As a result, an acidified solution of C_5_ and C_6_ sugars is obtained while water-soluble lignin species are removed from solution by the precipitation of lignin (Käldström et al., 2014a; Käldström et al., 2014b; Calvaruso et al., 2017a; Calvaruso et al., 2017b). In effect, MCD of lignocellulosic materials holds great promise as an entry-point process for integrated fractionation processes yielding sugars and lignins for the sustainable manufacture of chemicals and biofuels.

Despite the promise, progress in this exciting research area is still slowed down by difficulties in comparing results across the literature. When comparing reaction data, the description of milling conditions is insufficient to allow a direct analysis because the energy dose provided by a ball mill also depends on its mechanical design and grinding vessel geometry, which are often not provided. Therefore, to advance mechanochemistry in its breadth and depth, it is urgent to apply mathematical models to determine the energy dose provided by ball mills as a function of process parameters, thus generating a unified metric to analyze the reaction results.

Several studies have been dedicated to determining the kinetic energy dose that ball mills provide in metal alloying (Burgio et al., 1991; Magini et al., 1993; Magini et al., 1994; Magini et al., 1996). Despite the valuable insights gained in metal alloying, the application of kinematic modeling of ball mills to determine the energy dose provided by a mill to reactants in organic reactions is still in its infancy. Kinematic modeling of planetary ball mills provides the velocity and acceleration of a ball in a grinding jar, allowing for the determination of the energy dose supplied by a mill to a powder. Kinematic equations are derived from specific parameters of the mill mechanical designs, expressing the energy dose as a function of experimental parameters, *e.g.*, milling frequency, duration, ball count and size (Burgio et al., 1991; Magini et al., 1993; Magini et al., 1994; Magini et al., 1996). Based on the collision theory, the modeling considers the mill mechanical design of a mill in addition to the geometry of a grinding vessel. During the impacts on the jar walls, a ball transfers its kinetic energy to the powder. In the kinematic modeling, the impact events are taken as the dominant energy transfer mechanism. In this instance, friction is not accounted for in the kinematic equations of planetary mills. In practice, the operation conditions of a planetary mill are optimized by a trial-and-error approach to minimize friction, preventing excessive heat generation, which may cause the thermal decomposition of organic molecules and polymers.

We recently explored the concepts underlining the kinematic modeling to determine the energy dose provided by a high energy mill, shedding light on the macroscopic relations between energy dose and WSP yield obtained from the MCD of *α*-cellulose and beechwood acidified by H_2_SO_4_ impregnation (Kessler et al., 2018). Herein, we explore the energy dose as a unified explanatory variable to compare the results from MCD of beechwood acidified with H_2_SO_4_ performed in ball mills of different working principles under varied milling conditions. In this report, the presentation of results and discussion is organized into four parts. The first presents the mechanical design of the mills and the geometry of the grinding jars. The second describes the key aspects to consider in the kinematic modeling of planetary mills. Using energy dose as a unified metric of the milling parameters, the third part compares the results of 47 experiments performed in the planetary mill. The fourth part quantitatively examines two datasets obtained from different ball mill setups operating under varied milling conditions, providing detailed insight into the relationship between WSP yield and energy dose.

## Materials and Methods

### Materials

Beechwood chips (approximately 2 × 2 × 6 mm in dimension, Räuchergold^®^ KL 2–16 provided by J. Rettenmaier and Söhne), diethyl ether (for synthesis, VWR Chemicals); sulfuric acid (95%, VWR Chemicals) were used as received.

### Acidification of Beechwood by H_2_SO_4_ Impregnation

Beechwood (20 g) was suspended in a 0.125 mol/L sulfuric acid solution in diethyl ether (160 ml) under magnetic stirring (300 rpm) at room temperature for 2 h. Next, diethyl ether was removed under reduced pressure by using a rotary evaporator (Heidolph Rotary Evaporator Hei VAP series). The solvent-free, acid-impregnated substrate was immediately used in the MCD experiments.

### MCD Experiments Carried out in a Planetary Ball Mill

A planetary ball mill (Pulverisette seven premium line, Fritsch) equipped with stainless steel milling jars (80 ml filling capacity) and stainless steel balls were used. An experimental set was generated by varying rotational speed, milling time, and ball count and size. The milling parameters of the experiments are summarized in Supplementary Table S1 (Supplementary Material). The H_2_SO_4_-impregnated beechwood (6.4 g per jar) together with the defined number of milling balls were added to the jars and milled for the specified time. During the milling process, the jar temperature was held between 35 and 45°C by applying cooling cycles (in which the milling was paused until the jar cooled down to 35°C). The process times specified in Supplementary Table S1 apply exclusively to the milling with the set speed. The milled substrate was removed from the jars in a fume hood. WSP yield was determined directly in sequence.

### MCD Experiments Carried out in a High Energy Ball Mill

A high-energy ball mill (Emax mill, Retsch) equipped with stainless-steel milling jars (50 ml filling capacity) and stainless-steel balls were employed. An experimental set was generated by varying rotational speed, milling time, and ball count and size. The milling parameters of the experiments are listed in Supplementary Table S2 (Supplementary Material). The H_2_SO_4_-impregnated beechwood (4 g per jar) together with the defined number of milling balls were added to the jars and milled for the specified time. During the milling process, the jar temperature was controlled between 35 and 45°C by using an external cooling system attached to the mill (VWR RC-10 digital chiller). The process times specified in Supplementary Table S2 apply exclusively to the milling with the set speed. The milled substrate was removed from the jars in a fume hood. WSP yield was determined directly in sequence.

### Determination of WSP Yield

To a weighed centrifuge tube (50 ml Greiner Bio-One, polypropylene conical base centrifuge tube), the milled H_2_SO_4_-impregnated beechwood (1 g, weighed at a 0.1 mg precision) was added to the tube and suspended in distilled water (10 ml). The mixture was sonicated in an ultrasonic bath at room temperature for a total of 30 min (VWR USC 1200 HF ultrasonic bath operating at a frequency of 132 kHz). During the sonication, the centrifuge tube was manually shaken once in a while. Next, the mixture was centrifuged at 9,000 rpm (relative centrifugal force, RCF: 8,694) for 10 min in a Sigma 3–18 L centrifuge. The supernatant (WSP solution) was separated from the remnant solid. The centrifuge tube containing the insoluble residue was dried overnight at 40°C under reduced pressure (1 mbar). The weight of the centrifuge tube containing the dried solid residue was determined. The mass of solid residue ( $m_{solid\;residue}$ ) was determined by difference. WSP yield (%) was calculated by Eq. 1:

$$\mathrm{WSP}\left(\%\right)=\left(1-\frac{m_{\mathrm{solid residue}}}{m_{\mathrm{sample}}\mathrm{\times a}}\right)\times 100\;$$ (1)

Where: $m_{sample}$ stands for the mass of the milled sample added to the centrifuge tube, and a is the substrate-to-impregnated-substrate weight ratio (Meine et al., 2012).

### Digital Optical Microscopy and Particle Size Determination

Imaging and particle size determination was carried out on a Keyence VHX-5000 digital microscope. A total of 3,800–3,900 particles were measured. The particle size distribution was estimated from the maximum diameter of the particles.

## Results and Discussion

### Mechanical Design of the Mills and Geometry of the Grinding Jars

**FIGURE 1:**
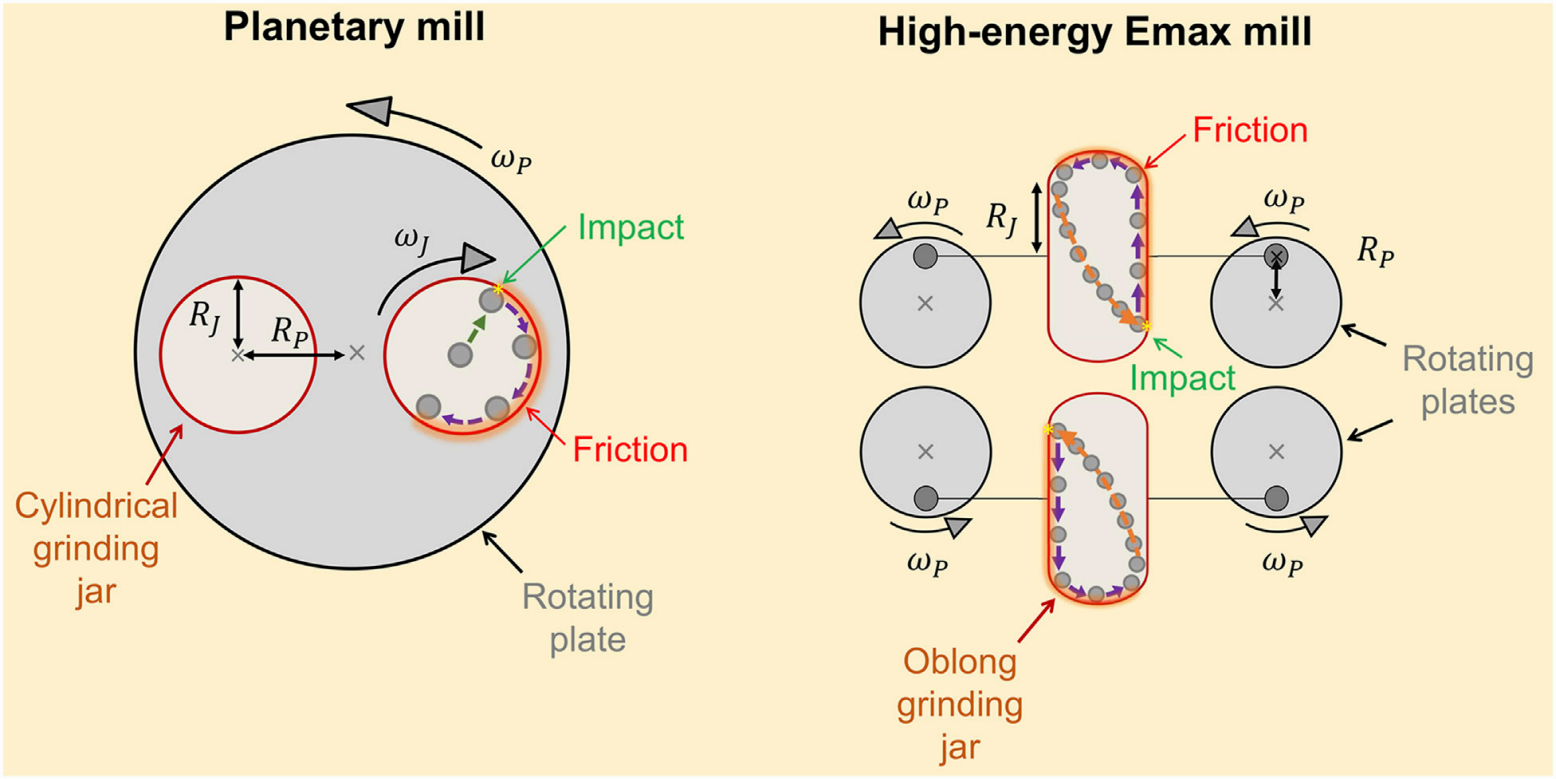
Simplified representation of the key features of the mechanical design of the planetary mill** (*left*)** and the high-energy Emax mill **(*right*)**. In this schematic representation, the trajectory of a ball for each grinding jar geometry is presented. The asterisk “*” in yellow color represents ball-wall impact points, and the blue arrows indicate the idealized moviment of the ball on the wall creating a ball-wall friction zone. For clarity, no representation of the grinding medium bulk is presented. However, ball-ball friction becomes substantial when loading the jar with a large number of balls of small diameter. Detailed information about the internal dimensions of the jars is presented in Supplementary Figure S1 (Supplementary Material).

The energy dose supplied by the mills depends on the mechanical design of the milling device and the geometry of the grinding jars (Burgio et al., 1991; Magini et al., 1993; Magini et al., 1994; Magini et al., 1996). In this section, we present the key features of the mechanical design of the planetary mill and the high-energy Emax mill. Figure 1 shows a simplified schematic representation of the two mills, indicating ball-wall impact points and ball-wall friction zones in the jar during the mill operation. The characteristic parameters of the two ball mills are listed in Table 1.

**TABLE 1:** Characteristic parameters of the planetary ball mill (“Pulverissete 7 Premium Line”) and the high-energy Emax ball mill (Retsch GmbH).

Parameter	Planetary mill	Emax mill
$R_{P}$ (mm)	70.0	16.0
$R_{J}$ (mm)	23.2	16.4
$\omega_{J}/\omega_{P}$	−2^[^ ^a^ ^]^	−1
Griding jar volume (ml)	80	50

a Parameter retrieved from: https://www.fritsch-international.com/sample-preparation/milling/planetary-mills/details/product/pulverisette-7-premium-line/

The planetary ball mill employs cylindrical grinding jars (Figure 1, *left*). The jars present an internal radius indicated by $R_{J}$ . They are placed on the rotating plate of the planetary mill. The distance between the rotating plate center and the jar center is defined as $R_{P}$ . During operation, the main plate rotates with an angular velocity $\omega_{P}$ defined by the rotational speed set by the operator. In addition, the jars rotate with an angular velocity $\omega_{J}$ (counter to the direction of the main plate). As defined by the manufacturer, the transmission ratio (also known as gear ratio, $\omega_{J}/\omega_{P}$ ) is equal to -2 for the “Pulverissete 7 Premium Line” (Table 1). Based on the mechanical design of the planetary mill, the balls move along with the inner jar wall (ball-wall friction zone) until they are launched against the opposite side of the jar by centrifugal forces (ball-wall impact point).

The high-energy Emax mill is designed to operate with oblong grinding jars (Figure 1, *right*). They are each fixed on two plates which are synchronously rotating with an $\omega_{P}$ value defined by the rotational speed set by the operator. $R_{P}$ stands for the distance from the plate center to the jar fixation point. During a milling process, the jars move on a circular path with no change in their orientation. Due to the oblong jar geometry and its controlled movement, the balls first roll and slide on the jar walls resulting in local, intensive friction. At the rounded jar ends, the balls undergo rapid acceleration and are then launched with high velocities against the opposing side of the jar (ball-wall impact point). As discussed in our previous work (Kessler et al., 2018) the parameter $R_{J}$ is approximated as half the length of the non-rounded section of the oblong-shaped jars. The ball mill manufacturer reported the transmission ratio $\omega_{J}/\omega_{P}$ to be equal to -1 for the high-energy Emax mill (Mahler and Pankratz, 2011).

### Kinematic Modeling of Planetary Mills

Burgio et al. (1991) and Magini et al. (1993), Magini et al. (1994), Magini et al. (1996) presented the kinematic modeling of planetary ball mills to determine the velocity of a ball $\left(v_{b}\right)$ launched against the jar walls during the mill operation. The kinetic energy of a ball ( $E_{b})$ can be determined by taking its mass ( $m_{b}$ ) into account, as given by Eq. 2:

$$E_{b}=\frac{1}{2}m_{b}v_{b}^{2}$$ (2)

Owing to momentum transfer, the ball velocity becomes $v_{S}$ after the impact with the inner jar wall. Hence, its residual energy ( $E_{S}$ ) is expressed as Eq. 3:

$$E_{S}=\frac{1}{2}m_{b}v_{S}^{2}$$ (3)

The energy transferred to the substrate from one ball during one collision ( $\Delta E_{b}$ ) is given by Eq. 4:

$$\Delta E_{b}=K_{a}\left(E_{b}-E_{S}\right)=\frac{1}{2}K_{a}m_{b}\left(v_{b}^{2}-v_{S}^{2}\right)$$ (4)

$K_{a}$ reflects the elasticity of the impact in which $K_{a}=1$ for inelastic collisions, and $K_{a}=0$ for elastic collisions (no energy will be transferred). Previous work showed that the impact of a ball covered with a substrate is almost perfectly inelastic, so that $K_{a}$ can be approximated with a value of 1 (Magini et al., 1993). Expressions for $v_{b}$ and $v_{S}$ depend on the absolute angular velocities of the main plate and the grinding jar ( $\omega_{P}$ and $\omega_{J}$ , respectively), the grinding jar radius ( $R_{J}$ ), the distance between the center of the main plate and the milling jar ( $R_{P}$ ), and the diameter of the milling balls used ( $d_{b}$ ), as presented in a great deal of detail elsewhere (Burgio et al., 1991). $\Delta E_{b}$ is given by Eq. 5:

$$\Delta E_{b}=-m_{b}\left[\frac{\omega_{J}^{3}\left(R_{J}-\frac{d_{b}}{2}\right)}{\omega_{P}}+\omega_{P}\omega_{J}R_{P}\right]\left(R_{J}-\frac{d_{b}}{2}\right)$$ (5)

However, the operation of planetary mills involves more than one ball. This implies that some balls will inevitably impede the impacts of others with the inner wall of the grinding jar. This reciprocal hindering depends on the degree of filling of the grinding jar. To account for the operation of the ball milling with multiple balls as the grinding medium, a yield coefficient ( $\varphi_{b}$ ) needs to be introduced in (5), rendering (6) that provides the energy dissipated by the impact of one ball against the grinding jar wall in a system with multiple balls (Burgio et al., 1991).

$$\Delta E_{b}^{*}=\varphi_{b}\;\Delta E_{b}$$ (6)

Burgio *et al.* derived the yield coefficient from analytical relations (Burgio et al., 1991). In short, $\varphi_{b}$ can be obtained from (7), where the parameter $n_{v}$ represents the ratio of total ball count **(** $N_{b})$ to the maximum ball count that can be contained in a cubic arrangement in the jar ( $N_{b,v})$ , as calculated from (8). In turn, $N_{b,v}$ is determined from (9), where $V_{J}$ and $d_{b}$ stand for the grinding jar volume and the ball diameter, respectively.

$$\varphi_{b}=1-n_{v}^{\varepsilon }$$ (7)

$$n_{v}=\frac{N_{b}}{N_{b,v}}$$ (8)

$$N_{b,v}=\frac{V_{J}}{d_{b}^{3}}$$ (9)

Finally, ε is a parameter depending on the ball diameter that can be estimated considering that less than one-third of the inner jar wall is covered with balls, a condition under which the reciprocal hindering is negligible. For this condition, $\varphi_{b}$ is analytically found to be equal to 0.95. By replacing this value in (7), the value of ε can be obtained by solving Eq. 10:

$${\left(\frac{N_{b,s}}{N_{b,v}}\right)}^{\varepsilon }=0.05$$ (10)

$N_{b,s}$ represents the number of balls needed to cover one-third of the inner jar wall in a cubic arrangement, as given by Eq. 11:

$$N_{b,s}=\frac{A_{J}}{3d_{b}^{2}}$$ (11)

$A_{J}$ is the “effective surface area” of the jar covered by the milling balls, where $A_{J}=\pi \left(D_{J}-d_{b}\right)H_{J}$ , with $H_{J}$ and $D_{J}$ corresponding to the jar height and diameter, respectively.

The collision frequency, $f_{b}$ , of one ball per unit time depends on the rotational speed during milling as given by Eq. 12:

$$f_{b}=\frac{K\left(\omega_{P}-\omega_{J}\right)}{2\pi }$$ (12)

Where K relates to the time required to dissipate the transferred energy. This parameter is canceled out when (12) is replaced in the energy equation of the kinematic modeling of planetary ball mills (13) (Burgio et al., 1991; Magini et al., 1996).

$$E=\frac{\varphi_{b}\Delta E_{b}N_{b}f_{b}t}{\mathrm{KW}}$$ (13)

where E stands for the energy dose given to a mass of substrate (*W*) in a milling process with duration t , including a certain number of milling balls, $N_{b}$ .

Replacing (5), (7) and (12) in (13), the expression for calculating the energy dose E becomes Eq. 14:

$$E=-\left(1-n_{v}^{\varepsilon }\right)N_{b}m_{b}t\left(\omega_{P}-\omega_{J}\right)\left[\frac{\omega_{J}^{3}\left(R_{J}-\frac{d_{b}}{2}\right)}{\omega_{P}}+\omega_{P}\omega_{J}R_{P}\right]\frac{\left(R_{J}-\frac{d_{b}}{2}\right)}{2\mathrm{\pi W}}$$ (14)

Finally, considering that $\omega_{J}=\;-2\omega_{P}$ for the planetary mill used in this study (Table 1, Fritsch, Pulverisette 7), Eq. 14 reduces to Eq. 15.

$$E=3\left(1-n_{v}^{\varepsilon }\right)N_{b}m_{b}t\omega_{P}^{3}\left[4\;\left(R_{J}-\frac{d_{b}}{2}\right)+R_{P}\right]\frac{\left(R_{J}-\frac{d_{b}}{2}\right)}{\mathrm{\pi W}}$$ (15)

The adaption of the kinematic model from a planetary ball mill to the high-energy Emax mill was detailed in our previous work (Kessler et al., 2018). To account for the Emax mill operation, the relation $\omega_{J}=\;-\omega_{P}$ was obtained from manufacturer patent (Mahler and Pankratz, 2011). Hence, (14) becomes (16), enabling estimating the energy dose provided by the high-energy Emax mill.

$$E=\left(1-n_{v}^{\varepsilon }\right)N_{b}m_{b}t\omega_{P}^{3}\left(R_{J}-\frac{d_{b}}{2}+R_{P}\right)\frac{\left(R_{J}-\frac{d_{b}}{2}\right)}{\mathrm{\pi W}}$$ (16)

### Energy Dose as a Unified Metric for Comparing Mechanochemical Reaction Results

**FIGURE 2:**
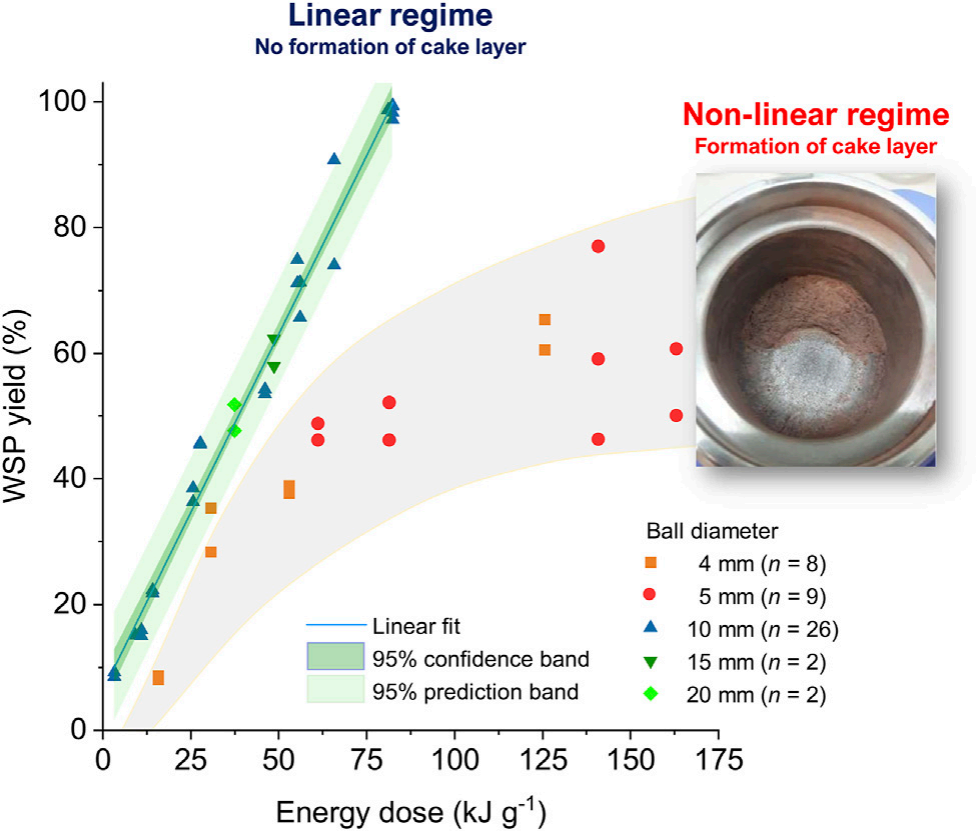
Dependence of WSP yield on the kinetic energy dose provided to H_2_SO_4_-impregnated beechwood by the planetary mill (Pulverisette seven premium, Fritsch) employing varied conditions as listed in Supplementary Table S1. Unlike the regions presented in green tones, which represent the 95% confidence band and 95% prediction band, the region highlighted in gray is solely intended to serve as a guide for the eyes, qualitatively outlining the data dispersion for the results obtained from MCD in the non-linear regime.

To examine the effect of the kinetic energy dose upon the WSP yield obtained from the MCD of H_2_SO_4_-impregnated beechwood, a total number (*n*) of 47 experiments were performed in a planetary mill operating under varied conditions, *i.e.*, different ball diameters (4–20 mm), ball counts (4–391), rotational speeds (400–800 rpm), and milling durations (0.18–4.4 h), as listed in Supplementary Table S1. Figure 2 presents a chart correlating the WSP yields with the respective energy doses provided by the planetary ball mill in each experiment.

From the WSP yield *vs.* energy dose chart, a linear and a non-linear regime for the dependence of WSP yield on the kinetic energy dose were identified for the MCD of H_2_SO_4_-impregnated beechwood. For the linear regime, WSP yields rose from 9% at 3.3 kJ g^−1^ to 99% at 81.1 kJ g^−1^. Notably, a low data dispersion was obtained, as indicated by the adjusted *r* ^2^ value (0.9834). Within the upper and lower 95% confidence bounds, the predicted values for the *mean* WSP yields are distributed ±2–3% around the fitted curve. In turn, within the upper and lower 95% prediction bounds, a new data point is expected to lie in a region ±7–8% around the fitted curve. The linear regime was observed when performing the MCD experiments employing a griding medium composed of balls of a diameter equal to or higher than 10 mm. For these (relatively) large balls, the overall ball surface area of the grinding medium is minimized. Hence, ball-ball friction occurring in the bulk of the grinding medium is reduced, ensuring a dominant energy transfer through the impact of the balls against the jar wall.

In stark contrast, no linear correlation was found in the highly scattered dataset obtained from the MCD of H_2_SO_4_-impregnated beechwood carried out using a grinding medium composed of balls of 4- or 5-mm in diameter. Interestingly, for the non-linear regime, WSP yields increased from 8% at 15.7 kJ g^−1^ to highly scattered values around 60% for energy doses between 125–165 kJ g^−1^. No further improvement in the WSP yields could be achieved by prolonging the milling duration or increasing the rotational speed. Hence, a limitation of the depolymerization extent (to a WSP yield of around 63%) is visible for the MCD of H_2_SO_4_-impregnated beechwood carried out employing a grinding medium composed of balls of 4- or 5-mm in diameter. The observation appears to be associated with the formation of a solid deposit (also known as cake layer or coating layer) on the grinding jar wall and/or grinding medium, as depicted in the inset of Figure 2. The cake layer has different mechanical properties than wood chips or sawdust in the initial milling stages of the H_2_SO_4_-impregnated beechwood. As a result, the formation of a thick layer of substrate buffers the ball-wall collision. The mechanism underlining the cake layer formation remains poorly understood in mechanochemistry. However, it is clear from Figure 2 that operating the ball mill for MCD in the linear regime, in which no cake layer is formed, corresponds to the best utilization of kinetic energy by the chemical reaction, boosting the WSP yield for a given energy dose provided by a ball mill.

### Comparison of MCD Results Obtained in Different Ball Mill Setups

**FIGURE 3:**
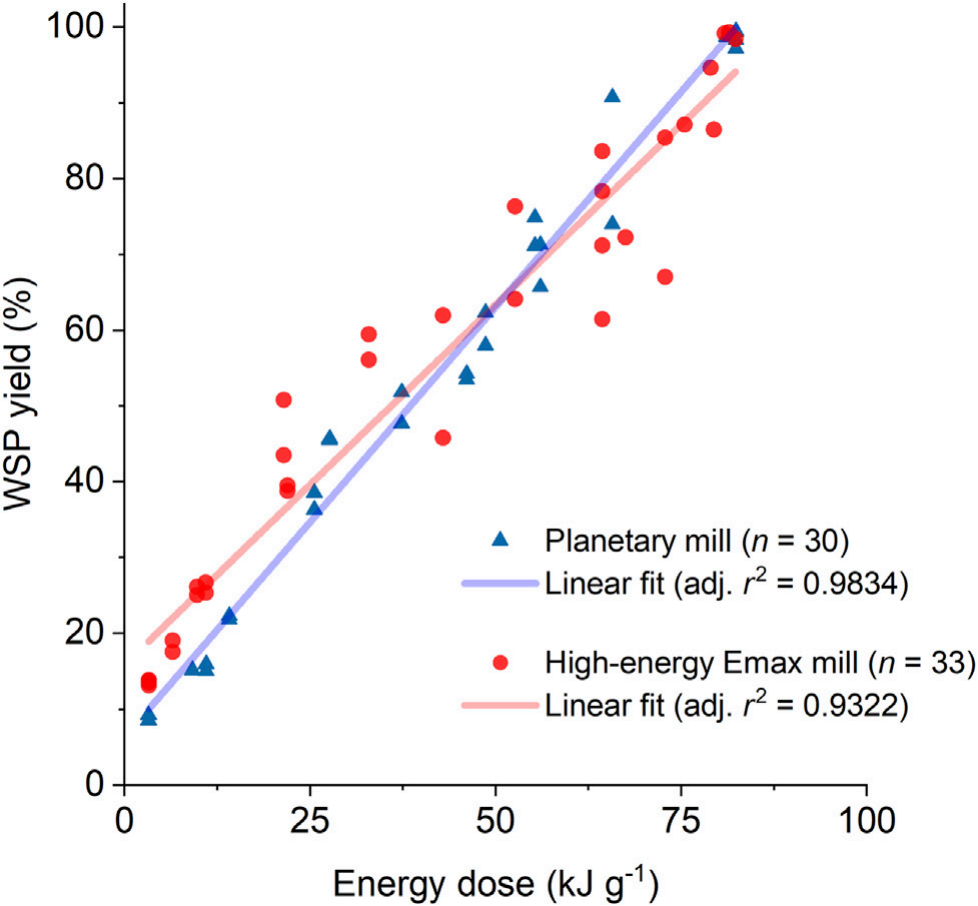
Comparison of MCD data from the experiments carried out in the high-energy Emax mill (Kessler et al., 2018) and the planetary ball mill (displaying the results from the linear regime) under varied conditions, and the linear fit for each dataset. Milling conditions are listed in Supplementary Table S1 and Supplementary Table S2.

The strong correlation between WSP yield and energy dose provided by the planetary mill indicates that the energy dose serves as a “compounded” explanatory variable, enabling a direct comparison of results obtained from experiments carried out in a planetary mill under varied experimental conditions. This section expands our analysis by comparing results obtained from ball mills of different operation principles. In this quest, we revisited the previously reported data obtained from the MCD of H_2_SO_4_-impregnated beechwood carried out in a high-energy Emax mill (Kessler et al., 2018). The high-energy Emax mill also produces a high-frequency impact of the griding medium against the jar walls like a planetary ball mill. Nonetheless, the operating mechanism of the high-energy Emax mill results in intensified frictional forces owing to the jar geometry and movement. The kinematic modeling of the ball mills solely includes the impact component as the energy transfer mechanism. In this manner, the comparison of MCD results obtained from the planetary mill and high energy Emax mill also provides qualitative but still critical insight into the effect of high friction on the MCD process. Figure 3 presents a chart comparing MCD data from the experiments carried out in the high-energy Emax mill or planetary ball mill (linear regime) under varied conditions and the linear fit for each dataset. The linear fit parameters are presented in Table 2.

**TABLE 2:** Linear regression parameters derived from the MCD datasets for the experiments carried out in the planetary and high-energy Emax mill, presented in Figure 3.

Parameter	Planetary mill	High-energy emax mill
Slope (%WSP yield·g_substrate_·kJ^−1^)	1.14 ± 0.03	0.95 ± 0.05
Intercept (%_WSP yield_)	6 ± 1	16 ± 2
Adjusted *r* ^2^	0.9834	0.9322
WSP productivity (g_WSP_ kJ^−1^)	0.0114 ± 0.0003	0.0095 ± 0.0005

For MCD of H_2_SO_4_-impregnated beechwood carried out in the high energy Emax mill, the WSP yields linearly increased with the energy dose provided by the mill (from 14% at 3.3 kJ g^−1^–99% at 81.5 kJ g^−1^). In stark contrast to the planetary mill experiments, no cake layer was formed in the high-energy Emax mill experiments, even when using a grinding medium composed of balls equal to or smaller than 5-mm in diameter. However, the results from the experiments carried out in the Emax mill presented a data dispersion larger than those from the experiments performed in the planetary mill, as indicated by the smaller adjusted *r* ^2^ value (0.9322 *vs.* 0.9834). *F*-test using the linear fit showed that the differences in the datasets are statistically relevant (*p* = 0.003). Likewise, the slopes are statistically different (*p* = 0.002). From the slopes obtained from the linear fitting, we derived the *WSP productivity* (expressed in g_WSP_ kJ^−1^) by multiplying the slope value by a factor of 0.01 (to convert the percentual yield into decimal yield), followed by algebraic simplification. For the MCD performed in the planetary mill, the WSP productivity was 20% higher than that found for the high-energy Emax mill (0.0114 ± 0.0003 g_WSP_ kJ^−1^ *vs.* 0.0095 ± 0.0005 g_WSP_ kJ^−1^, respectively). This difference is probably associated with the increased component of frictional forces provided by the Emax mill. Tentatively, the frictional forces would counteract the impact forces by realigning the polymer chains, thus reverting the conformational changes required for the activation of cellulose towards depolymerizing reactions (Schüth et al., 2014; Amirjalayer et al., 2019).

As also shown in Table 2, the intercept values are different [planetary mill: 6 ± 1% *vs.* high-energy Emax mill: 16 ± 2%). The intercepts present the WSP yield extrapolated at “time zero.” In the initial stage of the MCD process, it is natural to expect a redistribution of the acid catalyst impregnated in the beechwood chips when reducing the chip size from a millimetric down to a micrometric scale (Kaufman Rechulski et al., 2015). In effect, we expect the homogenization of acid catalyst distribution on the substrate to be a critical factor accounting for the difference in the intercept values, as the depolymerizing reactions on lignocellulose proceed very slowly when milling the substrate with no added acid catalyst. Notably, it takes over 1,000 h to achieve full conversion of cellulosic fibers into WSP by milling the substrate with no added catalyst] (Steurer and Hess, 1944; Grohn, 1958; Grohn and Schierbaum, 1958; Simionescu and Vasiliu-Oprea, 1969; Oprea, 1978).

**FIGURE 4:**
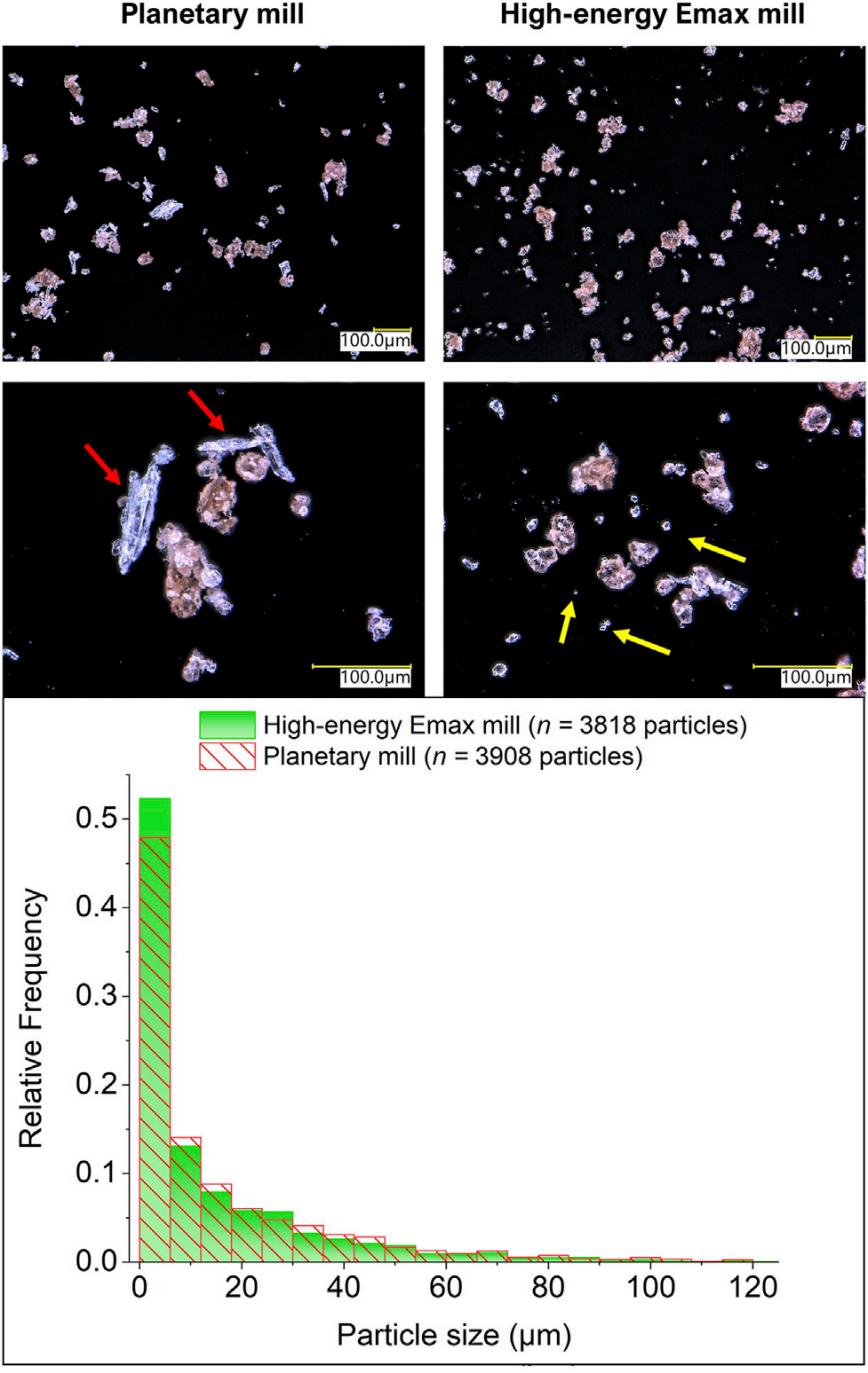
Digital optical micrographs and size particle distribution for H_2_SO_4_-impregnated beechwood processed at low energy dose (3.3 kJ mol^−1^) in different mills. The arrows in yellow color indicate small particles (1–6 μm) typically formed by the H_2_SO_4_-impregnated beechwood processed in a high-energy Emax mill. The arrows in red color indicate substantial residual particles (50–125 μm) typically obtained by processing H_2_SO_4_-impregnated beechwood in the planetary mill at a low energy dose.

To assess the impact of the different mills on the communition of H_2_SO_4_-impregnated beechwood chips, MCD was carried out in each ball mill operating under conditions to provide a low energy dose of 3.3 kJ mol^−1^. In sequence, the substrate was examined by digital optical microscopy at ×300 and ×800 magnification to determine the particle size distribution. Figure 4 displays the typical appearance of the particles and the particle size distribution obtained for each experiment.

Two classes of particles can be identified in the images in Figure 4. In the first, fibrous particles, which are white/translucent in appearance, are seen. In the second, small reddish-brown rugged aggregates are found. In detail, the powder product obtained in the planetary mill shows many large white fibrous particles next to the reddish-brown aggregates. In stark contrast, the powder product obtained in the high-energy Emax mill is a (relatively) homogeneous mixture of small reddish-brown aggregates with only very few fibrous white particles identified. In line with these qualitative observations, the particle distribution for the powder product obtained in the high-energy Emax mill shows a more prominent population of particle size smaller than 6 μm, compared to the particle size distribution for the powder product obtained in the planetary mill. In addition, the powder product obtained in the planetary shows populations of particles larger than 6 μm consistently higher than those present in the powder product from the high-energy Emax mill.

Overall, these observations substantiate the working hypothesis that the high-energy Emax mill reduces the size of beechwood chips more extensively than the planetary mill. As a result, we propose that the “initial stage” mass transfer process involved in the homogenization of the acid catalyst content on the substrate is more effective in the high-energy Emax mill, explaining the higher initial WSP yield for the MCD performed in the Emax mill at low energy doses. However, when the mass transfer limitations are relaxed, that is, when homogenization of the acid content is achieved, the WSP yield becomes governed by the intricacies of the energy dose transfer underlined by the operation principle of each ball mill setup.

## Conclusion

We demonstrated the energy dose as a unified metric to compare the results from MCD of H_2_SO_4_-impregnated beechwood performed in different ball mill setups operating under varied experimental parameters. The data comparison provided in-depth insight into the macroscopic relationships between the WSP yield and energy dose provided by a ball mill. Notably, linear correlations between WSP yields and energy dose were observed for the experiments carried out in both ball mills. These correlations show that the MCD carried out in a planetary mill presents a WSP productivity higher than that in a high-energy Emax mill by 20%. However, when a cake layer is formed on the grinding jar and/or the grinding medium, the productivity of WSP per energy dose markedly decreases. This observation indicates that the mechanochemical reactions should be performed under conditions that prevent the cake layer formation. Under such conditions, the best use of the energy dose by the chemical conversion is reached. Moreover, provided no cake layer is formed, we expect that the MCD of other types of lignocellulosic materials should also observe a linear correlation between WSP yield and energy dose, owing to the MCD process to be agnostic to the type of lignocellulosic substrate (Meine et al., 2012; Käldström et al., 2014a; Käldström et al., 2014b).

In the broader context, using the energy dose as a unified metric offers a productive avenue to understand the effects of mill setup and operational parameters upon the outcomes of mechano-assisted chemical reactions. Such data analysis is conducive to translating optimal process settings from one milling device to another via rational experiment design. In fact, beginning with the energy dose values required to achieve a certain chemical conversion extent, the macroscopic correlations between product yield and energy dose enable the translation of milling parameters between ball mills by employing the energy dose as an educated guess in the experiment design. Such a rational design of experiments holds promise to supersede the time-consuming trial-and-error approaches in the current practice of organic and inorganic syntheses assisted by mechanical forces.

## Data Availability

The original contributions presented in the study are included in the article/Supplementary Material, further inquiries can be directed to the corresponding author.
