# Supplementary material for: Kinetic Energy Dose as a Unified Metric for Comparing Ball Mills in the Mechanocatalytic Depolymerization of Lignocellulose
Source: Front Chem. 2022 Jan 3;9:816553. doi: 10.3389/fchem.2021.816553 (PMC8762196; doi:10.3389/fchem.2021.816553)
Supplement: Supplementary file 1 [file DataSheet1.docx]

Supplementary Material

Kinetic energy dose as a unified metric for comparing ball mills in the mechanocatalytic depolymerization of lignocellulose

Martin Kessler, Roberto Rinaldi*

Department of Chemical Engineering, Imperial College London, United Kingdom

*** Correspondence:** r.rinaldi1@imperial.ac.uk

**Contents**

Table S1. Milling conditions for experiments carried out in the planetary mill Pulverisette 7 premium line equipped with 80 mL grinding jars containing 6.4 g H_2_SO_4_-impregnated beechwood per jar.

Table S2. Milling conditions for experiments carried out in the high-energy Emax mill equipped with 50 mL grinding jars containing 4 g H_2_SO_4_-impregnated beechwood per jar.

Figure S1. Internal dimensions of the grinding jars of the planetary mill (Pulverisette 7 premium line, 80 mL jar) and the high-energy Emax mill (50 mL jar). Blue lines indicate the paths of friction.

Table S1: Milling conditions for experiments carried out in the planetary mill Pulverisette 7 premium line equipped with 80 mL grinding jars containing 6.4 g H_2_SO_4_-impregnated beechwood per jar.

| Entry | Ball diameter  (mm) | Ball count per jar  (unit) | Rotational speed  (rpm) | Milling time  (h) |
| --- | --- | --- | --- | --- |
| 1 | 4 | 391 | 400 | 1.5 |
| 2 | 4 | 391 | 500 | 1.5 |
| 3 | 4 | 391 | 600 | 1.5 |
| 4 | 4 | 391 | 800 | 1.5 |
| 5 | 5 | 201 | 500 | 4 |
| 6 | 5 | 201 | 500 | 8 |
| 7 | 5 | 201 | 600 | 4 |
| 8 | 5 | 261 | 600 | 1.5 |
| 9 | 10 | 33 | 600 | 1.5 |
| 10 | 10 | 34 | 500 | 0.5 |
| 11 | 10 | 34 | 500 | 1.17 |
| 12 | 10 | 34 | 500 | 3.75 |
| 13 | 10 | 34 | 600 | 1.73 |
| 14 | 10 | 26 | 500 | 1.5 |
| 15 | 10 | 26 | 500 | 0.5 |
| 16 | 10 | 26 | 500 | 3 |
| 17 | 10 | 26 | 400 | 1.5 |
| 18 | 10 | 26 | 500 | 4.4 |
| 19 | 10 | 26 | 500 | 2.5 |
| 20 | 10 | 26 | 500 | 0.18 |
| 21 | 15 | 10 | 600 | 1.5 |
| 22 | 20 | 4 | 600 | 1.5 |

Table S2. Milling conditions for experiments carried out in the high-energy Emax mill equipped with 50 mL grinding jars containing 4 g H_2_SO_4_-impregnated beechwood per jar.

| Entry | Ball diameter  (mm) | Ball count per jar  (unit) | Rotational speed  (rpm) | Milling time  (h) |
| --- | --- | --- | --- | --- |
| 1 | 2 | 2061 | 1500 | 3 |
| 2 | 3 | 611 | 1500 | 3 |
| 3 | 4 | 250 | 1500 | 3 |
| 4 | 5 | 128 | 1500 | 3 |
| 5 | 5 | 128 | 1300 | 3 |
| 6 | 6 | 74 | 1500 | 3 |
| 7 | 7 | 46 | 1500 | 3 |
| 8 | 8 | 31 | 1500 | 3 |
| 9 | 9 | 21 | 1500 | 3 |
| 10 | 10 | 16 | 1500 | 3 |
| 11 | 10 | 16 | 1500 | 2 |
| 12 | 10 | 16 | 1500 | 1 |
| 13 | 10 | 16 | 800 | 3 |
| 14 | 10 | 16 | 800 | 2 |
| 15 | 10 | 16 | 800 | 1 |
| 16 | 10 | 16 | 1200 | 3 |
| 17 | 10 | 16 | 1200 | 2 |
| 18 | 10 | 16 | 1200 | 1 |
| 19 | 10 | 16 | 1200 | 0.3 |


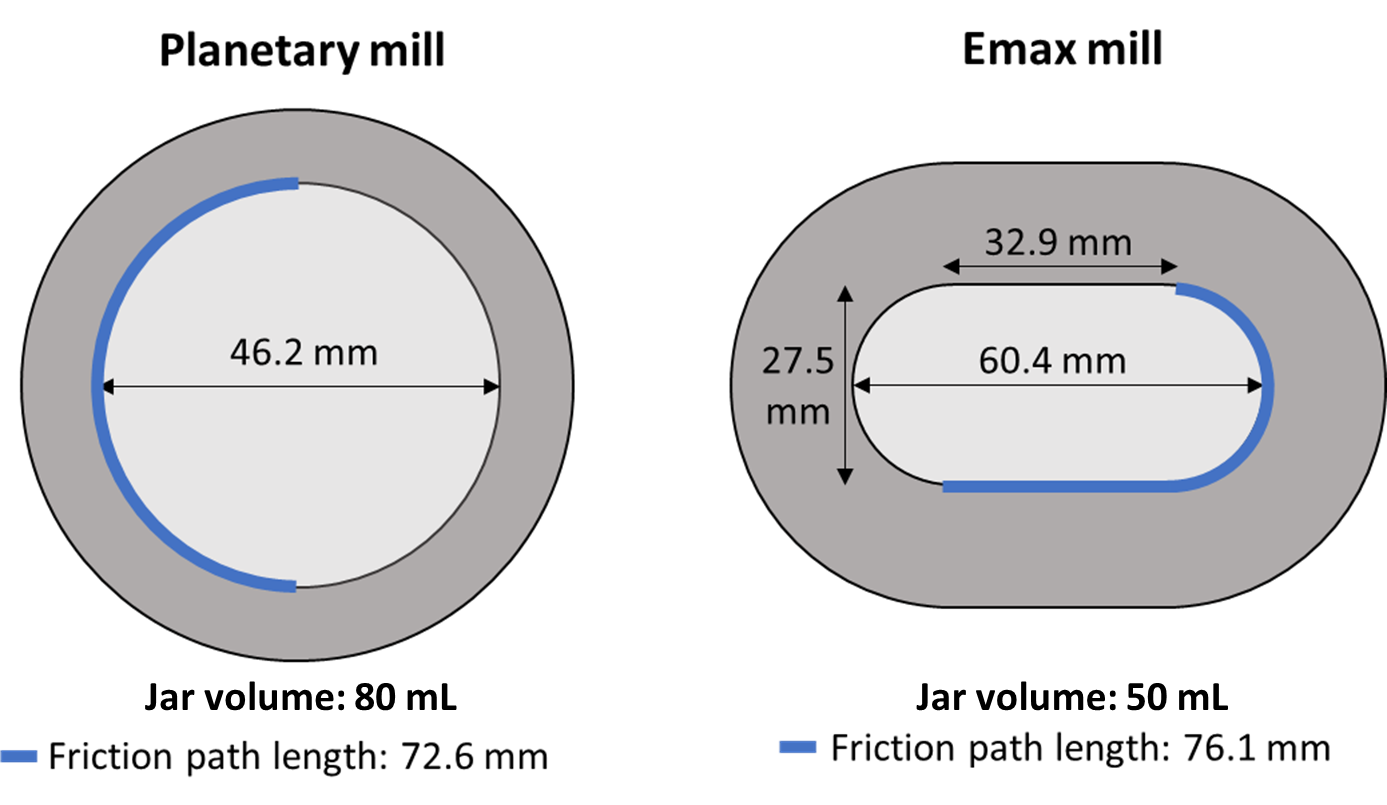


Figure S1. Internal dimensions of the grinding jars of the planetary mill (Pulverisette 7 premium line, 80 mL jar) and the high-energy Emax mill (50 mL jar). Blue lines indicate the paths of friction.
